# 1-Dec­yloxy-4-nitro­benzene

**DOI:** 10.1107/S160053680903921X

**Published:** 2009-10-03

**Authors:** Xi-Gui Yue

**Affiliations:** a Alan G. MacDiarmid Institute, Jilin University, Changchun 130012, People’s Republic of China

## Abstract

The title compound, C_16_H_25_NO_3_, has a zigzag dec­yloxy chain *para* to the nitro group of the aromatic ring. There are two independent mol­ecules; the two rings are aligned at 48.15 (7)°. In the crystal, weak C—H⋯O hydrogen bonds lead to the formation of infinite chains.

## Related literature

For structures of analogs of the title compound, see: McBurney *et al.* (2004 ▶).
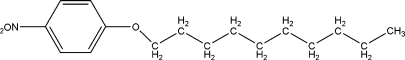


## Experimental

### 

#### Crystal data

C_16_H_25_NO_3_
*M* *_r_* = 279.37
Triclinic, 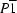

*a* = 5.642 (3) Å
*b* = 16.065 (8) Å
*c* = 19.135 (7) Å
α = 107.410 (16)°
β = 90.610 (16)°
γ = 99.780 (18)°
*V* = 1627.4 (13) Å^3^
*Z* = 4
Mo *K*α radiation
μ = 0.08 mm^−1^
*T* = 291 K
0.20 × 0.19 × 0.17 mm

#### Data collection

Rigaku R-AXIS RAPID diffractometer
Absorption correction: multi-scan (*ABSCOR*; Higashi, 1995 ▶) *T*_min_ = 0.984, *T*_max_ = 0.987
15990 measured reflections
7274 independent reflections
3970 reflections with *I* > 2σ(*I*)
*R*_int_ = 0.030

#### Refinement

*R*[*F* ^2^ > 2σ(*F* ^2^)] = 0.052
*wR*(*F* ^2^) = 0.176
*S* = 0.97
7274 reflections
363 parameters
H-atom parameters constrained
Δρ_max_ = 0.18 e Å^−3^
Δρ_min_ = −0.17 e Å^−3^

### 

Data collection: *RAPID-AUTO* (Rigaku, 1998 ▶); cell refinement: *RAPID-AUTO*; data reduction: *CrystalStructure* (Rigaku/MSC, 2002 ▶); program(s) used to solve structure: *SHELXS97* (Sheldrick, 2008 ▶); program(s) used to refine structure: *SHELXL97* (Sheldrick, 2008 ▶); molecular graphics: *Mercury* (Macrae *et al.*, 2006 ▶); software used to prepare material for publication: *SHELXL97*.

## Supplementary Material

Crystal structure: contains datablocks global, I. DOI: 10.1107/S160053680903921X/ng2644sup1.cif

Structure factors: contains datablocks I. DOI: 10.1107/S160053680903921X/ng2644Isup2.hkl

Additional supplementary materials:  crystallographic information; 3D view; checkCIF report

## Figures and Tables

**Table 1:** Hydrogen-bond geometry (Å, °)

*D*—H⋯*A*	*D*—H	H⋯*A*	*D*⋯*A*	*D*—H⋯*A*
C6—H6⋯O2^i^	0.93	2.53	3.378 (3)	152
C7—H7*B*⋯O5^ii^	0.97	2.77	3.263 (3)	112
C18—H18⋯O4^iii^	0.93	2.60	3.379 (3)	141
C21—H21⋯O1^iv^	0.93	2.70	3.346 (3)	127
C22—H22⋯O1^iv^	0.93	2.79	3.381 (3)	123

Symmetry codes: (i) 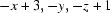; (ii) 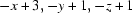; (iii) 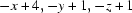; (iv) 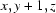.

**Fig. 1.**
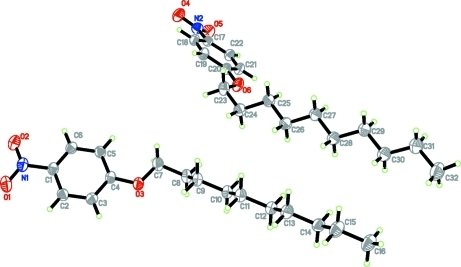
The asymmetric of title compound, with the atom numbering. Displacement ellipsoids of non-H atoms are drawn at the 30% probalility level.

## supplementary crystallographic information

### Comment

The hydroxy H atom of 4-nitropheno can be substituted by multifarious groups to form many ramifications(McBurney *et al.*, 2004). We have synthesized the analogs using different alkyl as terminal groups and report here the molecular and crystal structures of the title compound, (I).

The title compound, (I), as shown in Fig. 1, crystallizes in space group P-1 and each asymmetric unit consists of two crystallographically independent 1-(decyloxy)-4-nitrobenzene. The angles of the two benzene rings (C1—C6 and C17—C22) in the same asymmetric unit is 48.15 (7) °. All nitryl oxygen atoms are engaged in C—H···O (2.53 (2), 2.60 (2), 2.70 (2), 2.77 (2), 2.79 (2) Å) hydrogen bonds. The weak C—H···O hydrogen bonds link the crystal into a two-dimensional network.

### Experimental

1-(decyloxy)-4-nitrobenzene was prepared by adding 4-nitrophenol (0.14 g, 1 mmol), decyl iodide (0.27 g, 1 mmol) and acetone(15 ml) into 10 ml of 8% sodium hydroxide solution. The resultant mixture was heated for 2 h under reflux, then the solution was cooled to room temperaure in an ice bath with stirring. The colorless products were obtained by recrystallized the crude solid from 95% ethanol.

### Refinement

The benzene H atoms were treated as riding on their parent atoms, with C—H = 0.93 Å and *U*_iso_(H) = 1.2*U*_eq_(C). H atoms attached to methylene were treated as riding on their parent atoms with C—H = 0.97 Å and *U*_iso_(H) = 1.2*U*_eq_(C), instead with C—H = 0.96 Å and *U*_iso_(H) = 1.5*U*_eq_(C) for methyl.

### Crystal data

**Table:**

C_16_H_25_NO_3_	*Z* = 4
*M**_r_* = 279.37	*F*(000) = 608
Triclinic, *P*1	*D*_x_ = 1.140 Mg m^−^^3^
Hall symbol: -P 1	Mo *K*α radiation, λ = 0.71073 Å
*a* = 5.642 (3) Å	Cell parameters from 9752 reflections
*b* = 16.065 (8) Å	θ = 3.2–27.5°
*c* = 19.135 (7) Å	µ = 0.08 mm^−^^1^
α = 107.410 (16)°	*T* = 291 K
β = 90.610 (16)°	Block, colorless
γ = 99.780 (18)°	0.20 × 0.19 × 0.17 mm
*V* = 1627.4 (13) Å^3^	

### Data collection

**Table:**

Rigaku R-AXIS RAPID diffractometer	7274 independent reflections
Radiation source: fine-focus sealed tube	3970 reflections with *I* > 2σ(*I*)
graphite	*R*_int_ = 0.030
ω scans	θ_max_ = 27.5°, θ_min_ = 3.2°
Absorption correction: multi-scan (*ABSCOR*; Higashi, 1995)	*h* = −7→7
*T*_min_ = 0.984, *T*_max_ = 0.987	*k* = −20→20
15990 measured reflections	*l* = −23→24

### Refinement

**Table:**

Refinement on *F*^2^	Primary atom site location: structure-invariant direct methods
Least-squares matrix: full	Secondary atom site location: difference Fourier map
*R*[*F*^2^ > 2σ(*F*^2^)] = 0.052	Hydrogen site location: inferred from neighbouring sites
*wR*(*F*^2^) = 0.176	H-atom parameters constrained
*S* = 0.97	*w* = 1/[σ^2^(*F*_o_^2^) + (0.1*P*)^2^] where *P* = (*F*_o_^2^ + 2*F*_c_^2^)/3
7274 reflections	(Δ/σ)_max_ < 0.001
363 parameters	Δρ_max_ = 0.18 e Å^−^^3^
0 restraints	Δρ_min_ = −0.17 e Å^−^^3^

### Special details

**Table:**

Geometry. All e.s.d.'s (except the e.s.d. in the dihedral angle between two l.s. planes) are estimated using the full covariance matrix. The cell e.s.d.'s are taken into account individually in the estimation of e.s.d.'s in distances, angles and torsion angles; correlations between e.s.d.'s in cell parameters are only used when they are defined by crystal symmetry. An approximate (isotropic) treatment of cell e.s.d.'s is used for estimating e.s.d.'s involving l.s. planes.
Refinement. Refinement of *F*^2^ against ALL reflections. The weighted *R*-factor *wR* and goodness of fit *S* are based on *F*^2^, conventional *R*-factors *R* are based on *F*, with *F* set to zero for negative *F*^2^. The threshold expression of *F*^2^ > σ(*F*^2^) is used only for calculating *R*-factors(gt) *etc*. and is not relevant to the choice of reflections for refinement. *R*-factors based on *F*^2^ are statistically about twice as large as those based on *F*, and *R*- factors based on ALL data will be even larger.

### Fractional atomic coordinates and isotropic or equivalent isotropic displacement parameters (Å^2^)

**Table:**

	*x*	*y*	*z*	*U*_iso_*/*U*_eq_	
C1	1.0435 (3)	−0.10617 (12)	0.41928 (9)	0.0498 (4)	
C2	0.8180 (4)	−0.14170 (12)	0.38389 (10)	0.0552 (5)	
H2	0.7414	−0.1982	0.3826	0.066*	
C3	0.7089 (4)	−0.09201 (13)	0.35064 (10)	0.0561 (5)	
H3	0.5583	−0.1155	0.3258	0.067*	
C4	0.8218 (3)	−0.00675 (12)	0.35376 (9)	0.0492 (4)	
C5	1.0481 (3)	0.02798 (12)	0.38982 (9)	0.0518 (4)	
H5	1.1245	0.0848	0.3920	0.062*	
C6	1.1591 (3)	−0.02269 (13)	0.42251 (9)	0.0530 (5)	
H6	1.3112	−0.0002	0.4465	0.064*	
C7	0.7923 (4)	0.12696 (13)	0.32581 (11)	0.0622 (5)	
H7A	0.9486	0.1313	0.3052	0.075*	
H7B	0.8115	0.1628	0.3770	0.075*	
C8	0.6180 (4)	0.15878 (14)	0.28437 (10)	0.0608 (5)	
H8A	0.6685	0.2221	0.2934	0.073*	
H8B	0.4603	0.1494	0.3033	0.073*	
C9	0.5963 (4)	0.11315 (13)	0.20197 (9)	0.0557 (5)	
H9A	0.7539	0.1221	0.1828	0.067*	
H9B	0.5439	0.0499	0.1927	0.067*	
C10	0.4209 (4)	0.14703 (13)	0.16135 (10)	0.0562 (5)	
H10A	0.4705	0.2107	0.1724	0.067*	
H10B	0.2627	0.1363	0.1797	0.067*	
C11	0.4007 (4)	0.10524 (13)	0.07868 (10)	0.0575 (5)	
H11A	0.3507	0.0416	0.0674	0.069*	
H11B	0.5584	0.1161	0.0600	0.069*	
C12	0.2237 (4)	0.14028 (13)	0.03955 (10)	0.0569 (5)	
H12A	0.0659	0.1286	0.0579	0.068*	
H12B	0.2724	0.2041	0.0519	0.068*	
C13	0.2023 (4)	0.10103 (14)	−0.04315 (10)	0.0611 (5)	
H13A	0.1537	0.0372	−0.0557	0.073*	
H13B	0.3595	0.1130	−0.0618	0.073*	
C14	0.0239 (4)	0.13677 (14)	−0.08081 (10)	0.0591 (5)	
H14A	−0.1333	0.1241	−0.0624	0.071*	
H14B	0.0715	0.2007	−0.0673	0.071*	
C15	0.0008 (4)	0.10000 (17)	−0.16340 (11)	0.0763 (6)	
H15A	0.1577	0.1128	−0.1820	0.092*	
H15B	−0.0471	0.0360	−0.1771	0.092*	
C16	−0.1776 (5)	0.13638 (19)	−0.19974 (12)	0.0838 (7)	
H16A	−0.3362	0.1198	−0.1849	0.126*	
H16B	−0.1757	0.1127	−0.2521	0.126*	
H16C	−0.1349	0.1999	−0.1854	0.126*	
C17	1.6603 (3)	0.61062 (12)	0.48056 (9)	0.0483 (4)	
C18	1.7118 (3)	0.52982 (12)	0.44104 (9)	0.0515 (4)	
H18	1.8424	0.5094	0.4559	0.062*	
C19	1.5682 (3)	0.47936 (12)	0.37915 (9)	0.0528 (5)	
H19	1.6015	0.4246	0.3518	0.063*	
C20	1.3741 (3)	0.51059 (12)	0.35782 (9)	0.0485 (4)	
C21	1.3281 (4)	0.59306 (12)	0.39760 (10)	0.0550 (5)	
H21	1.2002	0.6145	0.3824	0.066*	
C22	1.4712 (3)	0.64329 (12)	0.45951 (10)	0.0549 (5)	
H22	1.4402	0.6985	0.4867	0.066*	
C23	1.2354 (4)	0.37683 (12)	0.25940 (10)	0.0599 (5)	
H23A	1.2308	0.3412	0.2924	0.072*	
H23B	1.3861	0.3757	0.2355	0.072*	
C24	1.0252 (4)	0.34081 (13)	0.20304 (10)	0.0597 (5)	
H24A	0.8776	0.3485	0.2278	0.072*	
H24B	1.0193	0.2776	0.1810	0.072*	
C25	1.0330 (4)	0.38395 (13)	0.14233 (9)	0.0577 (5)	
H25A	1.0412	0.4473	0.1641	0.069*	
H25B	1.1786	0.3752	0.1167	0.069*	
C26	0.8174 (4)	0.34758 (13)	0.08732 (10)	0.0596 (5)	
H26A	0.8071	0.2840	0.0667	0.072*	
H26B	0.6725	0.3575	0.1130	0.072*	
C27	0.8234 (4)	0.38781 (14)	0.02524 (10)	0.0636 (5)	
H27A	0.9663	0.3766	−0.0012	0.076*	
H27B	0.8377	0.4516	0.0459	0.076*	
C28	0.6052 (4)	0.35306 (15)	−0.02876 (11)	0.0664 (6)	
H28A	0.5915	0.2893	−0.0495	0.080*	
H28B	0.4625	0.3640	−0.0022	0.080*	
C29	0.6087 (4)	0.39286 (17)	−0.09064 (11)	0.0740 (6)	
H29A	0.7486	0.3802	−0.1181	0.089*	
H29B	0.6282	0.4568	−0.0699	0.089*	
C30	0.3882 (4)	0.36084 (16)	−0.14327 (11)	0.0727 (6)	
H30A	0.3688	0.2969	−0.1639	0.087*	
H30B	0.2485	0.3735	−0.1157	0.087*	
C31	0.3896 (5)	0.3997 (2)	−0.20473 (15)	0.1040 (10)	
H31A	0.5228	0.3834	−0.2343	0.125*	
H31B	0.4206	0.4638	−0.1842	0.125*	
C32	0.1645 (5)	0.3727 (2)	−0.25393 (14)	0.0943 (8)	
H32A	0.1262	0.3092	−0.2727	0.141*	
H32B	0.1884	0.3969	−0.2940	0.141*	
H32C	0.0344	0.3947	−0.2267	0.141*	
N1	1.1571 (3)	−0.15848 (12)	0.45537 (9)	0.0611 (4)	
N2	1.8046 (3)	0.66154 (12)	0.54848 (8)	0.0598 (4)	
O1	1.0497 (3)	−0.23246 (12)	0.45217 (11)	0.0953 (6)	
O2	1.3531 (3)	−0.12644 (11)	0.48909 (9)	0.0814 (5)	
O3	0.6957 (2)	0.03615 (9)	0.31962 (7)	0.0598 (4)	
O4	1.9796 (3)	0.63492 (11)	0.56572 (8)	0.0779 (5)	
O5	1.7400 (3)	0.72868 (11)	0.58678 (8)	0.0860 (5)	
O6	1.2164 (2)	0.46592 (9)	0.29918 (7)	0.0616 (4)	

### Atomic displacement parameters (Å^2^)

**Table:**

	*U*^11^	*U*^22^	*U*^33^	*U*^12^	*U*^13^	*U*^23^
C1	0.0558 (11)	0.0542 (11)	0.0446 (9)	0.0219 (9)	0.0075 (8)	0.0163 (7)
C2	0.0593 (12)	0.0479 (11)	0.0608 (11)	0.0114 (9)	0.0059 (9)	0.0190 (8)
C3	0.0517 (11)	0.0557 (12)	0.0607 (11)	0.0080 (9)	−0.0042 (9)	0.0188 (9)
C4	0.0543 (11)	0.0536 (11)	0.0428 (8)	0.0139 (8)	0.0003 (7)	0.0174 (7)
C5	0.0569 (11)	0.0500 (10)	0.0489 (9)	0.0059 (8)	−0.0016 (8)	0.0181 (8)
C6	0.0529 (11)	0.0605 (12)	0.0462 (9)	0.0129 (9)	−0.0013 (8)	0.0161 (8)
C7	0.0787 (14)	0.0553 (12)	0.0549 (10)	0.0099 (10)	−0.0063 (10)	0.0218 (9)
C8	0.0753 (14)	0.0561 (12)	0.0580 (10)	0.0216 (10)	0.0011 (9)	0.0228 (9)
C9	0.0632 (12)	0.0560 (11)	0.0547 (10)	0.0197 (9)	0.0011 (9)	0.0223 (8)
C10	0.0613 (12)	0.0558 (11)	0.0577 (10)	0.0188 (9)	0.0021 (9)	0.0223 (9)
C11	0.0623 (12)	0.0578 (12)	0.0558 (10)	0.0153 (9)	−0.0008 (9)	0.0204 (9)
C12	0.0581 (12)	0.0610 (12)	0.0553 (10)	0.0159 (9)	−0.0011 (9)	0.0205 (9)
C13	0.0654 (13)	0.0649 (13)	0.0536 (10)	0.0150 (10)	−0.0033 (9)	0.0171 (9)
C14	0.0568 (12)	0.0652 (13)	0.0553 (10)	0.0105 (10)	−0.0005 (9)	0.0190 (9)
C15	0.0738 (15)	0.0979 (18)	0.0552 (11)	0.0226 (13)	−0.0071 (10)	0.0168 (11)
C16	0.0803 (16)	0.113 (2)	0.0585 (12)	0.0234 (14)	−0.0106 (11)	0.0247 (12)
C17	0.0491 (10)	0.0482 (10)	0.0461 (9)	0.0050 (8)	0.0005 (7)	0.0142 (7)
C18	0.0495 (10)	0.0560 (11)	0.0525 (10)	0.0140 (8)	−0.0011 (8)	0.0198 (8)
C19	0.0611 (12)	0.0514 (11)	0.0466 (9)	0.0194 (9)	0.0010 (8)	0.0112 (8)
C20	0.0532 (11)	0.0513 (10)	0.0417 (8)	0.0115 (8)	−0.0008 (7)	0.0144 (7)
C21	0.0553 (11)	0.0543 (11)	0.0569 (10)	0.0189 (9)	−0.0033 (8)	0.0143 (8)
C22	0.0584 (11)	0.0458 (10)	0.0591 (10)	0.0156 (8)	0.0014 (9)	0.0106 (8)
C23	0.0765 (14)	0.0540 (11)	0.0481 (9)	0.0175 (10)	−0.0059 (9)	0.0112 (8)
C24	0.0676 (13)	0.0561 (11)	0.0509 (10)	0.0060 (9)	−0.0048 (9)	0.0130 (8)
C25	0.0613 (12)	0.0583 (12)	0.0503 (10)	0.0056 (9)	−0.0032 (9)	0.0150 (8)
C26	0.0608 (12)	0.0626 (12)	0.0521 (10)	0.0029 (10)	−0.0059 (9)	0.0173 (9)
C27	0.0603 (13)	0.0726 (14)	0.0571 (11)	0.0025 (10)	−0.0061 (9)	0.0239 (10)
C28	0.0628 (13)	0.0753 (14)	0.0598 (11)	−0.0001 (11)	−0.0096 (10)	0.0261 (10)
C29	0.0664 (14)	0.0923 (17)	0.0672 (12)	−0.0002 (12)	−0.0090 (11)	0.0387 (11)
C30	0.0696 (14)	0.0848 (16)	0.0630 (12)	0.0045 (12)	−0.0099 (10)	0.0272 (11)
C31	0.0847 (18)	0.150 (3)	0.0893 (17)	−0.0053 (17)	−0.0188 (14)	0.0705 (18)
C32	0.0967 (19)	0.118 (2)	0.0747 (15)	0.0257 (16)	−0.0117 (14)	0.0354 (14)
N1	0.0681 (11)	0.0645 (11)	0.0604 (9)	0.0272 (9)	0.0088 (8)	0.0251 (8)
N2	0.0583 (10)	0.0597 (11)	0.0558 (9)	0.0045 (8)	−0.0032 (8)	0.0128 (8)
O1	0.1013 (13)	0.0676 (11)	0.1324 (14)	0.0189 (9)	−0.0091 (11)	0.0523 (10)
O2	0.0739 (11)	0.0946 (12)	0.0890 (11)	0.0244 (9)	−0.0106 (9)	0.0433 (9)
O3	0.0613 (8)	0.0587 (8)	0.0645 (8)	0.0088 (6)	−0.0112 (6)	0.0279 (6)
O4	0.0709 (10)	0.0857 (11)	0.0717 (9)	0.0151 (8)	−0.0216 (8)	0.0163 (8)
O5	0.0878 (12)	0.0727 (11)	0.0754 (10)	0.0160 (9)	−0.0120 (8)	−0.0105 (8)
O6	0.0696 (9)	0.0574 (8)	0.0524 (7)	0.0203 (6)	−0.0148 (6)	0.0047 (6)

### Geometric parameters (Å, °)

**Table:**

C1—C6	1.371 (3)	C17—N2	1.462 (2)
C1—C2	1.381 (3)	C18—C19	1.378 (2)
C1—N1	1.454 (2)	C18—H18	0.9300
C2—C3	1.372 (3)	C19—C20	1.385 (3)
C2—H2	0.9300	C19—H19	0.9300
C3—C4	1.393 (3)	C20—O6	1.359 (2)
C3—H3	0.9300	C20—C21	1.385 (3)
C4—O3	1.354 (2)	C21—C22	1.375 (2)
C4—C5	1.388 (3)	C21—H21	0.9300
C5—C6	1.384 (2)	C22—H22	0.9300
C5—H5	0.9300	C23—O6	1.429 (2)
C6—H6	0.9300	C23—C24	1.507 (3)
C7—O3	1.437 (2)	C23—H23A	0.9700
C7—C8	1.501 (3)	C23—H23B	0.9700
C7—H7A	0.9700	C24—C25	1.518 (3)
C7—H7B	0.9700	C24—H24A	0.9700
C8—C9	1.521 (3)	C24—H24B	0.9700
C8—H8A	0.9700	C25—C26	1.514 (2)
C8—H8B	0.9700	C25—H25A	0.9700
C9—C10	1.516 (2)	C25—H25B	0.9700
C9—H9A	0.9700	C26—C27	1.511 (3)
C9—H9B	0.9700	C26—H26A	0.9700
C10—C11	1.517 (3)	C26—H26B	0.9700
C10—H10A	0.9700	C27—C28	1.513 (3)
C10—H10B	0.9700	C27—H27A	0.9700
C11—C12	1.517 (3)	C27—H27B	0.9700
C11—H11A	0.9700	C28—C29	1.504 (3)
C11—H11B	0.9700	C28—H28A	0.9700
C12—C13	1.513 (3)	C28—H28B	0.9700
C12—H12A	0.9700	C29—C30	1.507 (3)
C12—H12B	0.9700	C29—H29A	0.9700
C13—C14	1.513 (3)	C29—H29B	0.9700
C13—H13A	0.9700	C30—C31	1.487 (3)
C13—H13B	0.9700	C30—H30A	0.9700
C14—C15	1.508 (3)	C30—H30B	0.9700
C14—H14A	0.9700	C31—C32	1.494 (3)
C14—H14B	0.9700	C31—H31A	0.9700
C15—C16	1.506 (3)	C31—H31B	0.9700
C15—H15A	0.9700	C32—H32A	0.9600
C15—H15B	0.9700	C32—H32B	0.9600
C16—H16A	0.9600	C32—H32C	0.9600
C16—H16B	0.9600	N1—O1	1.222 (2)
C16—H16C	0.9600	N1—O2	1.223 (2)
C17—C22	1.373 (3)	N2—O4	1.220 (2)
C17—C18	1.374 (3)	N2—O5	1.225 (2)
			
C6—C1—C2	121.73 (17)	C17—C18—H18	120.3
C6—C1—N1	119.66 (17)	C19—C18—H18	120.3
C2—C1—N1	118.59 (18)	C18—C19—C20	119.66 (18)
C3—C2—C1	118.74 (18)	C18—C19—H19	120.2
C3—C2—H2	120.6	C20—C19—H19	120.2
C1—C2—H2	120.6	O6—C20—C19	124.99 (17)
C2—C3—C4	120.55 (18)	O6—C20—C21	114.94 (16)
C2—C3—H3	119.7	C19—C20—C21	120.08 (16)
C4—C3—H3	119.7	C22—C21—C20	120.23 (17)
O3—C4—C5	124.92 (17)	C22—C21—H21	119.9
O3—C4—C3	115.19 (16)	C20—C21—H21	119.9
C5—C4—C3	119.89 (17)	C17—C22—C21	118.89 (18)
C6—C5—C4	119.46 (17)	C17—C22—H22	120.6
C6—C5—H5	120.3	C21—C22—H22	120.6
C4—C5—H5	120.3	O6—C23—C24	107.35 (16)
C1—C6—C5	119.62 (17)	O6—C23—H23A	110.2
C1—C6—H6	120.2	C24—C23—H23A	110.2
C5—C6—H6	120.2	O6—C23—H23B	110.2
O3—C7—C8	107.42 (16)	C24—C23—H23B	110.2
O3—C7—H7A	110.2	H23A—C23—H23B	108.5
C8—C7—H7A	110.2	C23—C24—C25	114.59 (17)
O3—C7—H7B	110.2	C23—C24—H24A	108.6
C8—C7—H7B	110.2	C25—C24—H24A	108.6
H7A—C7—H7B	108.5	C23—C24—H24B	108.6
C7—C8—C9	114.15 (17)	C25—C24—H24B	108.6
C7—C8—H8A	108.7	H24A—C24—H24B	107.6
C9—C8—H8A	108.7	C26—C25—C24	113.38 (16)
C7—C8—H8B	108.7	C26—C25—H25A	108.9
C9—C8—H8B	108.7	C24—C25—H25A	108.9
H8A—C8—H8B	107.6	C26—C25—H25B	108.9
C10—C9—C8	113.08 (16)	C24—C25—H25B	108.9
C10—C9—H9A	109.0	H25A—C25—H25B	107.7
C8—C9—H9A	109.0	C27—C26—C25	114.50 (16)
C10—C9—H9B	109.0	C27—C26—H26A	108.6
C8—C9—H9B	109.0	C25—C26—H26A	108.6
H9A—C9—H9B	107.8	C27—C26—H26B	108.6
C9—C10—C11	114.62 (16)	C25—C26—H26B	108.6
C9—C10—H10A	108.6	H26A—C26—H26B	107.6
C11—C10—H10A	108.6	C26—C27—C28	114.52 (17)
C9—C10—H10B	108.6	C26—C27—H27A	108.6
C11—C10—H10B	108.6	C28—C27—H27A	108.6
H10A—C10—H10B	107.6	C26—C27—H27B	108.6
C10—C11—C12	113.45 (17)	C28—C27—H27B	108.6
C10—C11—H11A	108.9	H27A—C27—H27B	107.6
C12—C11—H11A	108.9	C29—C28—C27	115.03 (17)
C10—C11—H11B	108.9	C29—C28—H28A	108.5
C12—C11—H11B	108.9	C27—C28—H28A	108.5
H11A—C11—H11B	107.7	C29—C28—H28B	108.5
C13—C12—C11	114.79 (17)	C27—C28—H28B	108.5
C13—C12—H12A	108.6	H28A—C28—H28B	107.5
C11—C12—H12A	108.6	C28—C29—C30	115.36 (18)
C13—C12—H12B	108.6	C28—C29—H29A	108.4
C11—C12—H12B	108.6	C30—C29—H29A	108.4
H12A—C12—H12B	107.5	C28—C29—H29B	108.4
C12—C13—C14	113.73 (17)	C30—C29—H29B	108.4
C12—C13—H13A	108.8	H29A—C29—H29B	107.5
C14—C13—H13A	108.8	C31—C30—C29	115.8 (2)
C12—C13—H13B	108.8	C31—C30—H30A	108.3
C14—C13—H13B	108.8	C29—C30—H30A	108.3
H13A—C13—H13B	107.7	C31—C30—H30B	108.3
C15—C14—C13	115.11 (18)	C29—C30—H30B	108.3
C15—C14—H14A	108.5	H30A—C30—H30B	107.4
C13—C14—H14A	108.5	C30—C31—C32	115.9 (2)
C15—C14—H14B	108.5	C30—C31—H31A	108.3
C13—C14—H14B	108.5	C32—C31—H31A	108.3
H14A—C14—H14B	107.5	C30—C31—H31B	108.3
C16—C15—C14	114.2 (2)	C32—C31—H31B	108.3
C16—C15—H15A	108.7	H31A—C31—H31B	107.4
C14—C15—H15A	108.7	C31—C32—H32A	109.5
C16—C15—H15B	108.7	C31—C32—H32B	109.5
C14—C15—H15B	108.7	H32A—C32—H32B	109.5
H15A—C15—H15B	107.6	C31—C32—H32C	109.5
C15—C16—H16A	109.5	H32A—C32—H32C	109.5
C15—C16—H16B	109.5	H32B—C32—H32C	109.5
H16A—C16—H16B	109.5	O1—N1—O2	122.39 (18)
C15—C16—H16C	109.5	O1—N1—C1	118.43 (18)
H16A—C16—H16C	109.5	O2—N1—C1	119.15 (18)
H16B—C16—H16C	109.5	O4—N2—O5	122.67 (17)
C22—C17—C18	121.79 (16)	O4—N2—C17	119.36 (18)
C22—C17—N2	119.07 (17)	O5—N2—C17	117.95 (17)
C18—C17—N2	119.10 (17)	C4—O3—C7	118.14 (14)
C17—C18—C19	119.34 (17)	C20—O6—C23	118.71 (14)

### Hydrogen-bond geometry (Å, °)

**Table:**

*D*—H···*A*	*D*—H	H···*A*	*D*···*A*	*D*—H···*A*
C6—H6···O2^i^	0.93	2.53	3.378 (3)	152
C7—H7B···O5^ii^	0.97	2.77	3.263 (3)	112
C18—H18···O4^iii^	0.93	2.60	3.379 (3)	141
C21—H21···O1^iv^	0.93	2.70	3.346 (3)	127
C22—H22···O1^iv^	0.93	2.79	3.381 (3)	123

Symmetry codes: (i) −*x*+3, −*y*, −*z*+1; (ii) −*x*+3, −*y*+1, −*z*+1; (iii) −*x*+4, −*y*+1, −*z*+1; (iv) *x*, *y*+1, *z*.
